# Operieren, Absagen, Verschieben oder Selektionieren? Ergebnisse einer Umfrage unter gefäßchirurgischen Zentren während der Covid-19-Krise in Österreich

**DOI:** 10.1007/s00772-020-00686-5

**Published:** 2020-09-02

**Authors:** J. Klocker, A. Frech, A. Gratl, M. Thaler, I. Khosravi, M. Liebensteiner, M. Kluckner, W. Hofmann, A. Assadian, Österreichische Gesellschaft für Gefäßchirurgie (ÖGG), J. Klocker, M. Kluckner, W. Hofmann, A. Assadian

**Affiliations:** 1 Universitätsklinik für Gefäßchirurgie, Medizinische Universität Innsbruck, Anichstraße 35, 6020 Innsbruck, Österreich; 2 Universitätsklinik für Orthopädie, Medizinische Universität Innsbruck, Innsbruck, Österreich; 3 Abteilung für Gefäßchirurgie, LKH Feldkirch, Feldkirch, Österreich; 4 Abteilung für Gefäßchirurgie, Wilhelminenspital Wien, Wien, Österreich

**Keywords:** COVID-19, Gefäßchirurgie, OP-Verschiebung, OP-Triage, Umfrage, Severe acute respiratory syndrome coronavirus 2, Vascular surgical procedures, Surgery cancellation, Surgery triage, Survey

## Abstract

Gefäßchirurgische Zentren in Österreich (*n* = 15) wurden Mitte April 2020 mit erklärendem Begleitschreiben des Vorstands der Österreichischen Gesellschaft für Gefäßchirurgie (ÖGG) eingeladen, an einer Onlineumfrage über die Implikationen der COVID-19-Pandemie teilzunehmen. Insgesamt 12 Zentren (80 %) haben den Fragebogen ausgefüllt.

Alle Zentren waren mit positiv getesteten Patienten und 75 % auch mit positiv getesteten Mitarbeitern konfrontiert. Deutlich seltener war dies jedoch an den gefäßchirurgischen Abteilungen (positiv getestete Patienten in 25 %/Mitarbeiter in 33 %). Elektive Eingriffe wurden an allen Abteilungen entweder gestoppt (abgesagt oder verschoben) oder selektiv eingeschränkt. Dies betraf u. a. auch symptomfreie Patienten mit Karotisstenose, Aortenaneurysma kleiner 7 cm, peripherem Aneurysma, PAVK im Stadium II nach Fontaine oder Varikose. In allen Zentren wurden weiterhin uneingeschränkt gefäßchirurgische Notfälle behandelt. Uneinheitlich war das Vorgehen bei chronischen Ulzera, chronischer Mesenterialinsuffizienz, symptomfreiem Aortenaneurysma größer 7 cm und in der Shuntchirurgie.

Insgesamt war v. a. die zum Zeitpunkt der OP-Verschiebungen unklare Befristung der Maßnahmen problematisch, somit war auch das Risiko der abgesagten bzw. hinausgezögerten Behandlung nicht leicht abschätzbar. Für die Indikationen mit uneinheitlichem Vorgehen sollte unizentrisch und multizentrisch das Ergebnis der Patienten analysiert werden, auch hinsichtlich der mit der Verschiebung assoziierten psychologischen Belastung. Zudem bedürfen auch die aufgrund der COVID-19-Pandemie erfolgten Veränderungen im Alltag einer kritischen Auswertung, auch hinsichtlich demografischer und geografischer Unterschiede. Es ist anzunehmen, dass die Auswirkungen der COVID-19-Pandemie langfristig bedeutsam sind.

## Abstract (en)

Regional centers performing vascular surgery in Austria (*n* = 15) were invited in mid-April 2020 by the Austrian Society of Vascular Surgery (ÖGG) to participate in a nationwide survey about implications of the COVID-19 pandemic. Ultimately, a total of 12 centers (80%) answered the questionnaire.

All centers were confronted with patients who tested positive for COVID-19 and 75% also had medical personnel who were positive. In contrast, only 25% of the departments of vascular surgery had positively tested patients and 33% had positive staff members. In all departments of vascular surgery elective vascular procedures were either stopped (cancelled or deferred) or selectively limited, including patients with asymptomatic carotid stenosis, aortic aneurysms smaller than 7 cm, peripheral arterial aneurysm, peripheral artery occlusive disease Fontaine stage II and varicosities. All centers continued to carry out operations for all types of vascular surgical emergencies. The strategies of the centers were heterogeneous for patients with chronic ulcers, chronic mesenteric insufficiency, asymptomatic aortic aneurysms larger than 7 cm and shunt surgery.

Decisions on surgery cancellation seemed to be particularly problematic due to the uncertain time period of the COVID-19 measures. As a consequence, the risk associated with cancellation or delayed treatment was difficult to assess. At present, especially indications with nonuniform management strategies need selective attention and additional analysis in single center and multicenter studies. In addition, patients might suffer from relevant psychological problems because of surgery cancellations. Changes in the daily routine due to the COVID-19 pandemic may have a long-term impact on health status and may show significant demographic and geographic variations.

## Einführung

Bereits im Dezember 2019 wurde über eine in der Millionenmetropole Wuhan in China ausgebrochene Epidemie berichtet, die durch schwere Pneumonien und damit assoziierte Todesfälle charakterisiert und durch einen neuen Coronavirus (Severe Acute Respiratory Syndrome – Corona Virus 2, SARS-CoV-2) ausgelöst waren. Die nachfolgende weltweite Ausbreitung im Sinne einer Pandemie der dann Corona-Virus-Disease-19 (COVID-19) genannten Infektion erfolgte u. a. mit einem ersten europäischen Hotspot in Norditalien im Februar 2020 [2]. Aufgrund der geografischen Nähe Österreichs zu Norditalien mit häufiger medialer Berichterstattung über die äußerst problematische Situation im Nachbarland waren Behörden und Bevölkerung bereits erheblich alarmiert, als am 25. Februar 2020 erstmals in Österreich zwei Personen (italienischstämmige Hotelmitarbeiter in Innsbruck, beide kamen aus der Provinz Bergamo) positiv auf COVID-19 getestet wurden. Nachfolgend stieg die Anzahl der positiv auf COVID-19 getesteten Personen in Österreich rasch an, v. a. im Bundesland Tirol, sodass am 11. März 2020 die österreichische Bundesregierung die Schließung aller nicht systemnotwendigen Einrichtungen inkl. der Schulen und Universitäten beschloss. Am 12. März war dann der erste COVID-19-Todesfall in Österreich zu verzeichnen und ab 15. März 2020 wurde dann eine österreichweite generelle Ausgangsbeschränkung („Lockdown“) umgesetzt.

Zu diesem Zeitpunkt war allerdings unklar, wie viele der positiv auf COVID-19 getesteten Personen tatsächlich Krankheitssymptome entwickeln würden, wie viele davon in einem Krankenhaus behandelt und v. a. wie viele künstlich beatmet werden müssen. Es wurde anhand der damals vorliegenden Daten [4] davon ausgegangen, dass bis zu 14 % der positiv getesteten Patienten hospitalisiert werden müssen und bis zu 6 % einen Beatmungsplatz benötigen. Es war daher auch in Österreich schnell nötig, Ressourcen für Patienten mit schwerer COVID-19-Infektion freizumachen, indem nicht dringliche operative oder interventionelle Eingriffe vorläufig ausgesetzt (abgesagt oder verschoben) wurden. Insgesamt war die Einsparung limitierter Ressourcen hierbei aber nicht nur auf Bettenkapazitäten auf Normal- und Intensivstationen bezogen, sondern auch auf einzelne medizinische Verbrauchsgüter (z. B. OP- und FFP-Masken, Handschuhe, Desinfektionsmittel und Schutzkittel). Das „Fernhalten“ von Patienten mit nicht dringlichen bzw. nicht akuten Erkrankungen von Krankenhäusern diente zudem natürlich auch der Vermeidung einer potenziellen Exposition (COVID-19-positive Patienten bzw. Mitarbeiter).

Wie die Reduktion operativer oder interventioneller gefäßchirurgischer Eingriffe während der Phase des aufgrund der COVID-19-Pandemie ab 15. März 2020 angeordneten Lockdowns in Österreich erfolgte, welche gefäßchirurgischen Operationen weiter durchgeführt, abgesagt oder verschoben wurden, und wie einheitlich oder uneinheitlich dieses Vorgehen in verschiedenen gefäßchirurgischen Zentren des Landes war, wurde mittels eines Onlinefragebogens erfasst. Einzelne Aspekte des Fragebogens und der Ergebnisse werden im Folgenden dargestellt.

In Österreich gab es – zum Zeitpunkt unserer Umfrage – keine Stellungnahme der eigenen Fachgesellschaft (ÖGG), und auch die Österreichische Gesellschaft für Chirurgie hat für die gefäßchirurgischen Indikationen keine Empfehlungen zur Vorgehensweise in der Phase der COVID-19-Pandemie veröffentlicht.

## Umfrage mittels Onlinefragebogen in Österreich

Am 10. April 2020 wurden die Abteilungsleiter von insgesamt 15 österreichischen gefäßchirurgischen Zentren (Abteilungen mit ausschließlicher Ausrichtung auf Gefäßchirurgie oder chirurgische Abteilungen mit einem gefäßchirurgischen Schwerpunkt entsprechend dem Österreichischen Strukturplan Gesundheit/ÖSG 2017; alle in öffentlich-rechtlichen Zentral- oder Schwerpunktkrankenhäusern) per Mail mit erklärendem Begleitschreiben des Vorstands der Österreichischen Gesellschaft für Gefäßchirurgie (ÖGG) eingeladen, an einer kostenlosen anonymisierten Onlineumfrage über die aktuellen Implikationen der COVID-19-Pandemie auf die Patientenversorgung „in den letzten Wochen“ teilzunehmen. Die Umfrage wurde für Aspekte des Fachgebiets Gefäßchirurgie adaptiert aus einer zeitgleichen Befragung im Fachgebiet Orthopädie [5, 7] und mittels SurveyMonkey (http://www.surveymonkey.com), einem kostenlos verfügbaren Onlineumfragetool, erstellt. Da die Umfrage keinerlei Einzeldaten von Patienten betraf, war kein Votum einer Ethikkommission verpflichtend.

Insgesamt haben 12 von 15 (80 %) befragten gefäßchirurgischen Zentren den Onlinefragebogen in den Tagen zwischen 10. April bis spätestens 20. April beantwortet, wobei für die Auswertung nicht ersichtlich war, welche Zentren geantwortet haben. Auch war nicht ersichtlich, ob die Beantwortung durch die Abteilungsleiter selbst oder Mitarbeiter erfolgte. Die Auswertung erfolgte also verblindet und anonymisiert (dies wurde mit den einzelnen Abteilungen vorab so vereinbart).

Die Fragen waren entweder als Multiple-Choice (*n* = 9), als Einzelantwort (*n* = 4) oder als offene Fragen mit möglicher Textantwort (*n* = 2) formuliert und kreisten um 3 Themenbereiche: Implikationen der COVID-19-Pandemie und der hierzu durch die österreichischen Behörden erlassenen Bestimmungen („nationwide shutdown“) auf (1) das medizinische Fachpersonal, (2) die ambulante und stationäre Patientenversorgung, (3) die operative und interventionelle Patientenversorgung.

In der nachfolgenden Darstellung werden vornehmlich Aspekte der operativen und interventionellen Patientenversorgung behandelt, konkret, ob Eingriffe weiterhin generell oder lediglich selektiv durchgeführt, verschoben oder überhaupt abgesetzt wurden. Wie sich (im Nachhinein) herausgestellt hat, waren die Begriffe „abgesetzt“ und „verschoben“ schlecht zu differenzieren, wurden also vielfach nahezu synonym verwendet, da das „Absetzen“ einer OP (ein für alle Mal) selten anzunehmen ist und das „Verschieben“ allerdings in vielen Fällen mit unklarem und nicht festgelegtem Zeitfenster erfolgen musste, also de facto zunächst einem „Absetzen“ entsprach (zum Zeitpunkt der Befragung war die zeitliche Befristung der OP-Einschränkungen seitens der Behörden nicht absehbar).

Die zeitliche Befristung der OP-Einschränkungen war nicht absehbar

Entsprechend wurden auch im Onlinefragebogen die einzelnen Institutionen befragt, wie sie die Befristung der Einschränkungen der OP-Kapazitäten einschätzen. Hierbei wurden vordefinierte Zeitintervalle abgefragt: „5 bis 8 Wochen“, „9 bis 12 Wochen“, „3 bis 6 Monate“ oder „6 bis 9 Monate“.

Bereits mit Stichtag 22. April 2020, also bald nach der Schließung der Onlinebefragung, wurden vonseiten des Bundesministeriums für Soziales, Gesundheit, Pflege und Konsumentenschutz (BMSGPK) Empfehlungen zur schrittweisen Wiederaufnahme von aufgrund der COVID-19-Pandemie eingestellten bzw. reduzierten elektiven Tätigkeiten in Krankenanstalten herausgegeben, die dann aber in weiterer Folge erst schrittweise in den einzelnen Bundesländern Österreichs und auch mit regionalen Unterschieden umzusetzen waren.

## Ergebnisse der Umfrage – COVID-19-Fälle (Patienten und Mitarbeiter)

Alle Krankenhäuser waren mit positiv auf COVID-19 getesteten Patienten konfrontiert, jedoch hatten nur 25 % der gefäßchirurgischen Abteilungen positiv auf COVID-19 getestete Patienten zu behandeln. 75 % der Krankenhäuser hatten positiv auf COVID-19 getestete Mitarbeiter, allerdings nur 33 % der gefäßchirurgischen Abteilungen.

## Ergebnisse der Umfrage – OPs und Interventionen während des landesweiten Shutdowns

Alle Abteilungen berichteten, dass die Anzahl der während der COVID-19-Pandemie operierten Patienten drastisch reduziert wurde. Elektive Eingriffe wurden in allen gefäßchirurgischen Abteilungen entweder überhaupt gestoppt (für ambulante/tagesklinische OPs in 58 % der Zentren; für stationäre OPs in 42 % der Zentren) oder selektiv eingeschränkt (für ambulante/tagesklinische OPs in 42 % der Zentren; für stationäre OPs in 58 % der Zentren).

Die Befristung der Einschränkungen der OP-Kapazitäten (vordefinierte Zeitintervalle) haben die einzelnen Abteilungen sehr unterschiedlich eingeschätzt: „5 bis 8 Wochen“ (17 %), „9 bis 12 Wochen“ (33 %), „3 bis 6 Monate“ (17 %) oder „6 bis 9 Monate“ (33 %).

Zum Zeitpunkt der Umfrage berichtete keine Institution über fehlende Kapazitäten

Zum Zeitpunkt der Umfrage berichtete keine Institution über fehlende Kapazitäten bezüglich verfügbarer Betten (sowohl auf Normalstationen als auch auf Intermediate Care oder Intensivstationen) zur Behandlung von Patienten mit COVID-19-Infektion. Allerdings wurden in der Hälfte der Institutionen die den gefäßchirurgischen Abteilungen zugeordneten Bettenressourcen reduziert. Als Folge der antizipierten limitierten Ressourcen in der Gefäßchirurgie wurden folgende Pathologien nahezu einheitlich in allen Zentren gestoppt oder verschoben: symptomfreie Karotisstenose (Endarteriektomie: in 92 % der Zentren gestoppt oder verschoben; Karotisstent: in allen Zentren, die diese durchführen, gestoppt oder verschoben), symptomfreies Aortenaneurysma mit Maximaldurchmesser 5,5–7 cm (in 92 % der Zentren gestoppt oder verschoben), symptomfreie periphere Aneurysmen (in 92 % der Zentren gestoppt oder verschoben), periphere arterielle Verschlusskrankheit im Stadium II nach Fontaine (in allen Zentren gestoppt oder verschoben), Varikose (in allen Zentren gestoppt oder verschoben).

Im Gegensatz dazu wurden in allen gefäßchirurgischen Zentren *weiterhin uneingeschränkt* Patienten *operiert*, die eine akut behandlungsbedürftige arterielle Pathologie hatten: symptomatische Karotisstenose, akute Extremitätenischämie, symptomatisches oder rupturiertes Aneurysma (aortoiliakal oder peripher), akutes Aortensyndrom, traumatische Aortenruptur, akute Mesenterialischämie bzw. traumatische oder iatrogene Gefäßverletzung. Des Weiteren wurden in 92 % der Zentren in üblicher Weise weiterhin Patienten mit peripherer arterieller Verschlusskrankheit im Stadium III oder IV nach Fontaine behandelt, und auch weiterhin Major- und Minoramputationen durchgeführt. Auch wurden weiterhin in allen Institutionen zentralvenöse Katheter zur Hämodialyse und Portsysteme zur Chemotherapie angelegt.

*Uneinheitlich* war hingegen das Vorgehen der gefäßchirurgischen Zentren bei Patienten mit chronischen Ulzera (nur in 25 % weiterhin behandelt, in 75 % hingegen gestoppt bzw. verschoben), chronischer Mesenterialinsuffizienz (in 33 % weiterhin durchgeführt, in 67 % hingegen gestoppt bzw. verschoben) oder symptomfreiem Aortenaneurysma mit Maximaldurchmesser größer als 7 cm (in 75 % wurde eine offene oder endovaskuläre Behandlung weiterhin durchgeführt, in 25 % verschoben). Auch in der Shuntchirurgie war das Vorgehen sehr unterschiedlich: 50 % führten diese weiterhin durch, in den anderen 50 % der Zentren wurde diese jedoch gestoppt oder verschoben.

## Erkenntnisse der Umfrage

Die in Österreich in gefäßchirurgischen Zentren durchgeführte Onlineumfrage zeigt, dass in allen Institutionen Patienten mit COVID-19-Infektion behandelt wurden, aber vergleichsweise seltener Patienten und Mitarbeiter der gefäßchirurgischen Abteilungen betroffen waren. Das ist insbesondere auch deshalb bemerkenswert, da Patienten mit kardiovaskulären Erkrankungen, mit Diabetes oder mit arterieller Hypertonie als Hochrisikopatienten für eine fatal verlaufende COVID-19-Infektion gelten [6]. Sowohl Patienten als auch Gesundheitspersonal waren während der COVID-19-Pandemie einem vergleichsweise hohen und potenziell lebensbedrohlichen Risiko ausgesetzt, dies auch aufgrund einer mangelhaften Vorbereitung der Institutionen.

Die Institutionen waren auf die Pandemie nur unzureichend vorbereitet

Die COVID-19-Pandemie und der deshalb erfolgte Lockdown stellte auch in Österreich das Gesundheitspersonal und die Patienten vieler Krankenhausabteilungen, insbesondere auch jene der chirurgischen Fachgebiete, vor vielfältige Herausforderungen: u. a. mussten *elektive Indikationen* zur operativen und interventionellen Behandlung und deren Dringlichkeit eingehend *überprüft und hinterfragt* werden. Hierzu waren zum Zeitpunkt der Entscheidungsfindung für die Fachexperten aber keine Erfahrungsberichte vorliegend („welche Operationen wie lange verschieben?“), das Zeitfenster für Verschiebungen war völlig unklar (einige Monate? ein halbes Jahr? noch länger?) und das Risiko für den Patienten aufgrund einer abgesagten bzw. hinausgezögerten Behandlung in vielen Fällen nicht leicht abschätzbar.

Entsprechend haben auch in der Beantwortung des Onlinefragebogens die einzelnen Institutionen die *unklare Befristung der Einschränkungen* der OP-Kapazitäten sehr unterschiedlich eingeschätzt. Konkret nahmen 50 % der Institutionen an, dass diese zumindest 3 Monate oder länger andauern würde. Zum Zeitpunkt der Entscheidung zur OP-Verschiebung wurde die Befristung also (mit heutigem Wissen ex post) länger eingeschätzt, als sie tatsächlich war, da angenommen werden kann, dass viele Zentren ab Mai 2020 wieder zu einer „normalen Performance“ zurückkehren konnten.

Interessant scheint in diesem Zusammenhang die Publikation eines auf einer Expertenumfrage basierten Modells, das für die aktuelle COVID-19-Pandemie mittels Bayes’scher linearer Regression abschätzte, wie viele Fallabsagen elektiver Operationen weltweit innerhalb von 12 Wochen „angenommenem Stillstand“ (für Elektivoperationen) zu erwarten wären: über 28 Mio. Operationen bzw. 72,3 % aller Operationen wären betroffen. Selbst wenn – nach der COVID-19-Pandemie – die OP-Kapazitäten um 20 % gesteigert werden, würde die Aufarbeitung der verschobenen Operationen median 45 Wochen in Anspruch nehmen. Aus gefäßchirurgischer Sicht ist an dieser Publikation der *COVIDSurg Collaborative* zu kritisieren, dass (1) Europa und Zentralasien gemeinsam ausgewertet wurden (trotz anzunehmender beträchtlicher regionaler Unterschiede), und (2) die Gefäßchirurgie in der Kategorie „other specialties“ gemeinsam mit der Herzchirurgie, Thoraxchirurgie, Neurochirurgie und Mammachirurgie (!) dargestellt wurde [3].

Unsere Onlineumfrage zeigt aber auch, dass die *Entscheidung* zum Verschieben bzw. Absetzen von gefäßchirurgischen Operationen und Interventionen in den unterschiedlichen Institutionen *in vielen Fällen einheitlich* erfolgte, gewissermaßen „nach einem logischen Konzept“: Notfälle und dringliche Fälle wurden weiterhin ohne Verzögerung behandelt, elektive Eingriffe regelhaft postponiert. Allerdings war die Entscheidung *für einzelne Krankheitsbilder bzw. Indikationen uneinheitlich*, u. a. die Shuntchirurgie (Shuntanlage bei funktionierendem HD-Katheter?), das Management von Patienten mit chronischen Ulzera (Revaskularisation bzw. Major- oder Minoramputationen bei annähernd stabilem Fontaine Stadium IV? Revaskularisation bei nicht unmittelbar drohendem Beinverlust?) oder chronischer Mesenterialinsuffizienz (Revaskularisation bei stabiler Angina abdominalis?). Zudem war auch das Vorgehen bei symptomfreiem Aortenaneurysma uneinheitlich: während nahezu alle Institutionen symptomfreie Aortenaneurysmen kleiner als 7 cm verschoben oder absetzten, wurden Aneurysmen mit Maximaldurchmesser größer 7 cm in 75 % der Institutionen weiterhin behandelt.

Die Onlineumfrage war für die „Indikationen mit uneinheitlichem Management“ möglicherweise zu wenig detailliert, d. h. eine weitere Ausformulierung der Fragen hätte vielleicht mehr Klarheit geschaffen. Jedenfalls sind die „Indikationen mit uneinheitlichem Management“ wohl jene *Themenbereiche, die künftig einer weiteren Festlegung bedürfen*, und somit auch einer weiteren *Forschungsaktivität* (z. B. über den natürlichen Krankheitsverlauf), um herauszufinden, wie hier insgesamt besser selektioniert werden kann.

In der bisher publizierten Literatur finden sich u. a. *Triage-Empfehlungen* für die Gefäßchirurgie erstellt vom *American College of Surgeons* (ACS) [1]: diese empfehlen hinsichtlich der o. g. „Indikationen mit uneinheitlichem Management“ interessanterweise eine Intervention zu verschieben für Patienten mit symptomfreiem Aortenaneurysma kleiner als 6,5 cm („postpone“), aber auch für jene größer als 6,5 cm („postpone if possible“). Auch wird empfohlen, die Behandlung bei CLTI (Stadium III oder IV nach Fontaine) oder bei chronischer Mesenterialinsuffizienz falls vertretbar zu verschieben (jeweils „postpone if possible“). Hinsichtlich der Shuntchirurgie wird detailliert differenziert: Von einer Shuntneuanlage wird abgeraten („consider postponing“), auch von einer Shuntrevision bzw. Shuntangiographie bei schlechter Funktion („postpone if possible“), jedoch wird eine Notfallintervention bei thrombosiertem Shunt bzw. Nichtfunktion, bei Steal oder bei Infekt empfohlen. Diese Empfehlungen des American College of Surgeons (ACS) stellen wohl in erster Linie einen Expertenkonsensus dar, jedenfalls wird keine Literatur als Beleg bzw. kein Evidenzlevel angegeben.

Eine in der Lockdown-Phase durchgeführte Onlineumfrage liefert naturgemäß keine Daten über die klinischen Ergebnisse der Patienten, sowohl jener, die behandelt wurden, als auch jener, deren Operation oder Intervention abgesetzt bzw. verschoben wurde. Diese Daten wären wichtig, v. a. für die abgesetzten bzw. verschobenen Patienten, werden aber schwer zu erheben sein (die Kohorte besteht auch aus den Patienten, die aufgrund der COVID-19-Pandemie niemals ärztlich untersucht wurden, oder jenen mit Komplikationen, die vielleicht auch ohne die COVID-19-Pandemie aufgetreten wären) und werden – falls überhaupt – wohl erst zu einem deutlich späteren Zeitpunkt (in 6 bis 12 Monaten?) der „Aufarbeitung“ der verschobenen Fälle erfasst werden können.

Die Onlineumfrage liefert keine Daten über die klinischen Ergebnisse der Patienten

Nicht zuletzt muss auch auf die – unabhängig von klassischen kardiovaskulären Ergebnisparametern – potenzielle zusätzliche *psychologische Belastung* der Patienten hingewiesen werden, die durch OP-Absagen und OP-Verschiebungen eingetreten ist. Die unklar befristete „Wartezeit“, potenziell mögliche Komplikationen währenddessen und die Auswirkung der Verunsicherung auch auf die Lebensqualität der Patienten sind sicherlich nicht zu unterschätzen. Zusätzlich ungünstig könnte sich ausgewirkt haben, dass in der Wartezeit auch die Arzt-Patienten-Kommunikation nicht in üblicher Art und Weise erfolgen konnte, da die Patienten seitens der Gesundheitsbehörden aufgefordert wurden, „sich von Krankenhäusern nach Möglichkeit fern zu halten“.

## Ausblick

Aufgrund der COVID-19-Pandemie erfolgte OP-Verschiebungen und Veränderungen im Alltag der Patienten (Verhaltensänderungen betreffend Lifestyle inkl. körperliche Aktivitäten und Psyche, zu erwartende Auswirkungen auf Risikofaktoren wie Rauchen, Alkoholkonsum und Diät) sollten hinsichtlich ihrer gesundheitlichen Auswirkungen nicht unterschätzt werden. Zu erwarten ist, dass diese Auswirkungen demografische und geografische Unterschiede zeigen, dass sich langfristig wirksame positive und negative gesundheitliche Konsequenzen zeigen und dass diese abhängig von den angeordneten Maßnahmen und deren zeitlicher Befristung sind. Alle diese Aspekte erfordern eine auf die Problemstellung angepasste und somit neuartige kritische wissenschaftliche Analyse inklusive der Einbeziehung neuer Konzepte im Alltag (z. B. visuelle und supportive Telemedizin), insbesondere auch in der Gefäßmedizin. Es gilt also wohl längerfristig das auf die COVID-19-Pandemie bezogene Zitat „we are only at the beginning of this journey together“ [6].

## Fazit für die Praxis

- Die COVID-19-Pandemie traf – sicherlich auch in Österreich – ein auf eine derartige Krise nur eingeschränkt vorbereitetes Gesundheitssystem.
- Entscheidungsträger, auch im Fachgebiet Gefäßchirurgie, waren gefordert, die Kapazitäten für Akutfälle und dringliche Fälle aufrecht zu erhalten, mussten aber Verschiebungen elektiver Operationen (mit primär unklarem Zeitfenster) anordnen.
- Für einzelne Indikationen (z. B. symptomfreies Aortenaneurysma größer als 7 cm, chronische Ulzera o. chronische Mesenterialischämie, Shuntchirurgie) war das Vorgehen während der COVID-19-Pandemie in den befragten österreichischen Institutionen uneinheitlich.
- Diese Bereiche sollten hinsichtlich der klinischen Ergebnisse nach OP-Verschiebungen, der Möglichkeiten zur detaillierteren Selektion und der Strategien zur Komplikationsvermeidung weiter analysiert und beforscht werden.
